# NRF1 mitigates motor dysfunction and dopamine neuron degeneration in mice with Parkinson's disease by promoting GLRX m^6^A methylation through upregulation of METTL3 transcription

**DOI:** 10.1111/cns.14441

**Published:** 2023-09-22

**Authors:** Xin Gong, Mengyi Huang, Lei Chen

**Affiliations:** ^1^ Department of Neurosurgery, Hunan Provincial People's Hospital The First Affiliated Hospital of Hunan Normal University Changsha Hunan P.R. China

**Keywords:** dopamine neuron degeneration, glutaredoxin, IGF2BP2, m^6^A methylation, methyltransferase‐like 3, motor dysfunction, nuclear factor erythroid 2‐like 1, Parkinson's disease

## Abstract

**Objective:**

The feature of Parkinson's disease (PD) is the heavy dopaminergic neuron loss of substantia nigra pars compacta (SNpc), while glutaredoxin (GLRX) has been discovered to modulate the death of dopaminergic neurons. In this context, this study was implemented to uncover the impact of GRX1 on motor dysfunction and dopamine neuron degeneration in PD mice and its potential mechanism.

**Methods:**

A PD mouse model was established via injection with 1‐methyl‐4‐phenyl‐1,2,3,6‐tetrahydropyridine (MPTP) into mice. After gain‐ and loss‐of‐function assays in mice, motor coordination was assessed using rotarod, pole, and open‐field tests, and neurodegeneration in mouse SNpc tissues was determined using immunohistochemistry of tyrosine hydroxylase and Nissl staining. NRF1, methyltransferase‐like 3 (METTL3), and GLRX expression in SNpc tissues were evaluated using qRT‐PCR, Western blot, and immunohistochemistry. The N6‐methyladenosine (m^6^A) levels of GLRX mRNA were examined using MeRIP. The relationship among NRF1, METTL3, and GLRX was determined by RIP, ChIP, and dual luciferase assays.

**Results:**

Low GLRX, METTL3, and NRF1 expression were observed in MPTP‐induced mice, accompanied by decreased m^6^A modification level of GLRX mRNA. GLRX overexpression alleviated motor dysfunction and dopamine neuron degeneration in MPTP‐induced mice. METTL3 promoted m^6^A modification and IGF2BP2‐dependent stability of GLRX mRNA, and NRF1 increased METTL3 expression by binding to METTL3 promoter. NRF1 overexpression increased m^6^A modification of GLRX mRNA and repressed motor dysfunction and dopamine neuron degeneration in MPTP‐induced mice, which was counteracted by METTL3 knockdown.

**Conclusion:**

Conclusively, NRF1 constrained motor dysfunction and dopamine neuron degeneration in MPTP‐induced PD mice by activating the METTL3/GLRX axis.

## Graphical abstract

NRF1 promotes m6A modification and IGF2BP2‐dependent stability of GLRX mRNA by increasing the expression of METTL3 and leads to the increase of GLRX expression, thus alleviating motor dysfunction and dopamine neuron degeneration in Parkinson's disease mice.

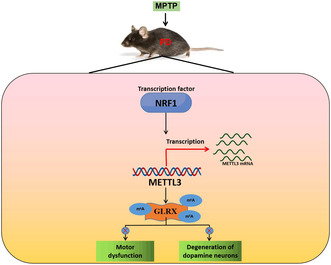

## 1 INTRODUCTION

Parkinson's disease (PD), a prevalent neurodegenerative disease, is mainly featured by dopaminergic neuron death in the substantia nigra pars compacta (SNpc).^1^, ^2^ PD is related to the following risk factors, including age (the most prominent risk factor), genetics, environmental factors (such as insecticides and water contaminants), and behavioral factors (such as coffee, tobacco use, head trauma, or exercise) and more susceptible to males than females.^3^ This disease is related to motor (such as rigidity, postural instability, bradykinesia, and resting tremor) and non‐motor (including fatigue, pain, autonomic dysfunction, olfactory dysfunction, psychiatric symptoms, cognitive impairment, and sleep disturbances) features.^4^, ^5^ Cures are shelved and eventually result in death since PD is generally identified at a late stage of complete neuronal degeneration because of the absence of early diagnostic technologies.^6^ Therefore, the search for molecules deregulated in PD may shed new light on the diagnosis and treatment of PD.

Glutaredoxin (GLRX), a small protein with one active site cysteine pair, can catalyze glutathione‐dependent redox modulation through glutathionylation, glutathione conjugation to the substrate, and deglutathionylation.^7^ GLRX is vital for maintaining the intracellular reduced environment and protecting against oxidative stress, which exerts a critical function in the pathology of most neurodegenerative diseases.^8^ Miller et al. elaborated that GLRX1 regulates apoptotic signaling in dopaminergic neurons and loss of GLRX1 results in enhanced cell death in PD.^9^ Likewise, knockdown of GLRX led to augmented apoptosis in immortalized dopaminergic neurons.^10^

N6‐methyladenosine (m^6^A), a methylation occurred at the adenosine N6 position, represents the commonest chemical modification of eukaryotic mRNAs, which is installed by methyltransferases, removed by demethylases, and recognized by reading proteins to modulate RNA metabolism, such as translation, splicing, folding, export, degradation, and mRNA stability.^11^, ^12^ Methyltransferase‐like 3 (METTL3) is a m^6^A methyltransferase,^13^ which was predicted to be proportional to GLRX expression in SNpc by GEPIA2 website in our research. m^6^A methylation is a critical RNA modification abundant in cerebral tissues, and the genes engaged in m^6^A modification are associated with diverse neurological disorders.^14^ A prior article identified the downregulation of m^6^A modifications of mRNAs in the brain striatum of PD rats and 6‐OHDA‐induced PC12 cells.^15^ Of note, METTL3 is downregulated in human Alzheimer's disease brain.^16^

METTL3 expression in SNpc was also predicted to be associated with nuclear factor erythroid 2‐like 1 (NRF1) expression by GEPIA2 website. NRF1, a transcription factor that belongs to the Cap'N' Collar family, is a commonly expressed endoplasmic reticulum transmembrane protein engaged in detoxification and stress adaptation, as well as transcription regulation via genomic antioxidant response elements.^17^ NRF1 is an essential transcription regulator of proteasomal gene expression in neurons, and disruption of NRF1 function may cause neurodegenerative disease pathogenesis.^18^ Furthermore, NRF1 has been reported to be downregulated in PD rats.^19^ Therefore, it was reasonable to conjecture that the NRF1/METTL3/GLRX axis might influence PD progression. The present study was expected to delve into the impact of NRF1, METTL3, and GLRX on PD and the underlying mechanisms.

## 2 MATERIALS AND METHODS

### 2.1 Establishment of a PD mouse model and stereotactic injection

Eight‐week‐old male C57BL/6 mice (weighing 22–25 g, Shanghai SLAC Laboratory Animal Co., Ltd.) were housed in separate cages in a specific pathogen‐free animal laboratory at 22–25°C with 60%–65% humidity and free access to water and food under a 12‐h cycle of light and darkness. Experiments were initiated after 1 week of acclimatization with the health status of mice inspected before the experiments. All experiments on animals abided by the regulations and codes of practice for laboratory animal management and the ethical requirements of laboratory animals and were ratified by the animal care and use committee of Hunan Provincial People's Hospital.

Eight mice were randomly assigned to each group. Each mouse in the PD group was intraperitoneally injected with 30 mg/mL 1‐methyl‐4‐phenyl‐1,2,3,6‐tetrahydropyridine (MPTP, 30 mg/kg, Sigma‐Aldrich) dissolved in 0.9% sterile normal saline for 5 days. Mice in the control group were injected with an equal amount of normal saline in the same manner as the negative control (NC). Behavioral experiments were conducted 1 week later.

For adeno‐associated virus (AAV) injection, mice in the PD group were anesthetized with a mixture of isoflurane/oxygen 2 weeks prior to MPTP injection.^20^ After the mice were fixed on a stereotactic device (David Kopf Instrument), the skin over the skull was incised and drilled with a surgical drill to expose the skull. A 10 μL Hamilton syringe (33‐gauge needle) was utilized for the injection with 2 μL AAV serotype 9 solution (5 × 10^13^ vg/mL) at a rate of 0.2 μL/min. The injections were given at anterior–posterior −3.60, dorsal‐ventral (DV) −3.75, and medio‐lateral +1.15. After injection, the syringe was left in place for 5 min and then slowly withdrawn. Behavioral tests to assess motor function‐related behaviors in mice were performed 8 weeks after injection.^21^

The target sequence was cloned into pAAV‐CAG‐enhanced green fluorescent protein (EGFP) vector (28014, Addgene) and validated by sequencing. AAV‐GFP virus was produced and purified by Hanbio. AAV titers were determined by quantitative real‐time polymerase chain reaction (qRT‐PCR), accompanied by the measurement of virus purity: AAV‐GFP (titer: 4.7 × 10^14^ vg/mL), AAV‐GLRX (titer: 5.7 × 10^14^ vg/mL), AAV‐GFP (titer: 6.1 × 10^14^ vg/mL), AAV‐short hairpin RNA (sh)NC (titer: 7 × 10^14^ vg/mL; 5′‐TTTGTTGGTTACGGGGTATCGATTCAAGAGATCGATACCCCGTAACCAACTTTTT‐3′), and AAV‐shMETTL3 (titer: 3.7 × 10^14^ vg/mL; 5′‐TTTGCGGATGCAGTGATCTAATAAGTTCTCTATTAGATCACTGCATCCGCTTTTT‐3′).^22^

### 2.2 Rotarod test

The rotarod test was applied to test the motor coordination of mice. Before the formal test, mice were trained twice a day for 3 days. On the fourth day, the mice were placed on a rotarod to measure the duration of stay. The rotation speed was accelerated from 0 to 30 rpm within 1 minute, followed by 5‐min test at 30 rpm. The test was completed when the mice fell off the rotarod or grabbed and did not walk on the rotarod after the rotarod was rotated twice in a row. The test data were counted as the time the mice spent moving on the rotarod.^23^

### 2.3 Pole test

The mice were trained on a climbing pole for 3 days, twice a day. The test setup consisted of non‐slip metal rod with a length of 50 cm and a diameter of 1.2 cm and a ball with a diameter of 2 cm at the top of the pole. The pole was placed at an angle of 45° to the horizontal. Mice were placed face up on the ball, followed by the recording of the total time from the time mice stood on the ball to the time they climbed to the bottom of the pole.^23^

### 2.4 Hanging test

The hanging test was performed to examine the neuromuscular strength and motor function of mice. Briefly, the test setup consisted of a box and a metal bar with a diameter of 12 mm. The metal bar was placed horizontally at a height of 30 cm with a lid 1 cm above the bar to prevent the mice from sitting on the bar. During the test, the timing was started after the mice were hung on the metal bar and stopped when the mice fell. The test was repeated three times for each mouse, at an interval of 1 min, after which the mean hanging time of each mouse was counted. The test was repeated three times individually.^24^

### 2.5 Open‐field test

The open‐field test, or spontaneous activity, was a common indicator for detecting hypermobility after MPTP injury. The size of the test boxes was 500 × 500 × 300 mm, and the color of the perimeter wall was black. The bottom of the open field was evenly divided into 16 small squares of 4 × 4. The camera was set up directly above the test box, with the field of view covering the entire open field. The animals were placed in the central square, followed by videography and timing for 5 min. The activity status of mice during a certain period was analyzed using a computerized tracer analysis system. The laboratory was kept quiet, with around 20°C room temperature and sufficient light. The observation indexes were the number of crossings between the squares (the limbs of mice entered from one square into another square as one pass) and the total movement distance. The test was repeated three times individually.

### 2.6 Brain tissue acquisition

After the behavioral test, the mice were anesthetized, followed by the insertion of a perfusion needle from the left ventricle into the aorta. Normal saline was injected to flush out the blood, and the heart was injected with 4% paraformaldehyde to completely immobilize the brain. The brain was extracted and fixed in paraformaldehyde for 48 h. The midbrain was excised, dehydrated in gradient ethanol, cleared in xylene, embedded in paraffin, and sectioned to identify the SNpc region, followed by preparation of 5 μm brain sections for use.^23^

### 2.7 Immunohistochemistry and immunofluorescence

Sections were dewaxed with xylene and rehydrated with gradient alcohol.

For immunohistochemistry, the sections were incubated with 3% hydrogen peroxide to block endogenous peroxidase activity. Slides underwent 30‐min boiling in 10 mM sodium citrate (pH 6.0) and 15‐min sealing in 10% normal goat serum. Slides were incubated overnight with antibodies (1:100, Invitrogen) against GLRX (PA5‐92389), NRF1 (MA5‐32782), METTL3 (PA5‐121190), or tyrosine hydroxylase (TH, PA1‐4679) in a wet chamber at 4°C. The following day, slides were washed with phosphate‐buffered saline (PBS) and incubated with secondary antibodies at room temperature for 1 h. Immunoreactivity was measured with the diaminobenzidine (DAB) kit (Invitrogen), followed by observation and photographing under an inverted microscope. ImageJ software was employed for analysis.^23^, ^24^

For immunofluorescence, the sections were treated with citrate buffer (0.1 M, pH 6.0) for 10 min at 95°C for antigen repair. Following three washes with PBS with 0.2% Tween‐20 for 10 min each, the sections underwent 10‐min treatment with 0.5% Triton X‐100, sealing in 5% bovine serum albumin, and overnight incubation with TH antibodies (PA1‐4679, 1:100, Invitrogen) in a wet chamber at 4°C. Then, the sections were incubated with Alexa Fluor 546‐coupled goat anti‐rabbit antibodies (1:500, Invitrogen) and Hoechst 33342 (1:1000, Life Technologies) for 1 h, washed thrice with PBS, and sealed with anti‐fluorescence quenching agent. Five different fields were chosen under the FV‐1000/ES confocal microscope for observation and photography.^24^

### 2.8 Nissl staining

The sections underwent 2‐h baking at 65°C, dewaxing with xylene, rehydration with gradient alcohol, and staining with preheated Nissl staining solution (Beyotime) at 56°C for 10 min. Thereafter, the sections were subjected to color separation for several seconds and rinsed with double‐distilled water, followed by twice treatment with anhydrous ethanol and xylene, respectively. Following being mounted with neutral gum, the sections were observed and photographed with an inverted microscope, and the Nissl body was stained in purple.^23^

### 2.9 TdT‐mediated dUTP‐biotin nick end‐labeling

Apoptosis was determined with the TdT‐mediated dUTP‐biotin nick end‐labeling (TUNEL) kit (C1091, Beyotime) as per the specifications. Paraffin sections were dewaxed in xylene for 5–10 min, dewaxed again in fresh xylene for 5–10 min, and treated with anhydrous ethanol for 5 min, 90% ethanol for 2 min, 70% ethanol for 2 min, and distilled water for 2 min. The sections were reacted with 20 μg/mL DNase‐free proteinase K (ST532, Beyotime) for 15–30 min at 20–37°C, and washed thrice with PBS. The sections underwent 20‐min incubation with PBS‐prepared 3% hydrogen peroxide solution at room temperature, three PBS washes, and 60‐min incubation with 50 μL TUNEL solution at 37°C in the dark. After that, the sections were subjected to 30‐min incubation with Streptavidin‐horseradish peroxidase working solution at room temperature, and development with DAB (each step was followed by three PBS washes). After the sections were mounted, five different fields were chosen under the inverted microscope for observation, photography, and analysis.

### 2.10 Cell culture and transfection

Mouse microglia BV‐2 cells (Procell) underwent cultivation in minimum essential medium with 10% fetal bovine serum and 1% penicillin/streptomycin (Gibco) at 37°C with 5% CO_2_. Small interfering RNAs (siRNAs) were synthesized by Hanbio Biotechnology: si‐NC (5′‐UUCUCCGAACGUGUCACGUTT‐3′), si‐METTL3 (5′‐GGACCAAGGAAGAGUGCAU‐3′), si‐insulin‐like growth factor 2 mRNA‐binding protein 1 (si‐IGF2BP1: 5′‐GCAAGCUAUCAUGAAGCUATT‐3′), si‐IGF2BP2 (5′‐GCAGAGAAGCCUGUCACAATT‐3′), si‐IGF2BP3 (5′‐GCAGAGGAUUCGUAAACUUTT‐3′), and si‐NRF1 (5′‐CACAUUGGCUGAUGCUUCAUU‐3′), as well as oe‐GLRX and oe‐NC. Transfection was conducted using Lipofectamine 2000 reagent (Invitrogen), and follow‐up experiments were carried out after 48‐h transfection.

### 2.11 Quantitative real‐time polymerase chain reaction

Total cell or tissue RNA was isolated with TRIZOL (Invitrogen), followed by reverse transcription as per the manuals of a reverse transcription kit (TaKaRa). Gene expression was examined using a LightCycler 480 fluorescent quantitative PCR instrument (Roche Diagnostics). The reaction conditions were set as per the protocols of the fluorescent quantitative PCR kit (SYBR Green Mix, Roche Diagnostics), including 10 s at 95°C, 45 cycles of 5 s at 95°C, 10 s at 60°C, and 10 s at 72°C, and 5‐min extension at 72°C. Three replicates were set for each quantitative PCR. The internal reference was β‐actin, and the 2^−ΔΔCt^ method was applied for data analysis. The primers are specified in Table 1.

**TABLE 1:** Primer sequences.

Name of primer	Sequences (5′–3′)
GLRX	F: TCCTCAGTCAACTGCCTTTCA
	R: CTCCGGTGAGCTGTTGTAAA
METTL3	F: CCCAACCTTCCGTAGTGATAG
	R: TGGCGTAGAGATGGCAAGAC
NRF1	F: TCGTGGGTGGTAGGGTACAT
	R: TCTAGCAGAGGTCTAGGCGG
METTL14	F: GGTCGGAGTGTGAACCTGAT
	R: GGTCCTCTTCCACGCTGTAT
IGF2BP1	F: CTTTGTAGGGCGTCTCATTGGC
	R: CCTTCACAGTGATGGTCCTCTC
IGF2BP2	F: TGAAGCCTGTGCCAATGCTGAG
	R: CCAGTCGAAAAGATGCCAAGTGC
IGF2BP3	F: CCACCCAGTTTGTTGGAGCCAT
	R: GGATAGTAATGGACTTCTCCGCG
β‐Actin	F: CATTGCTGACAGGATGCAGAA
	R: ATGGTGCTAGGAGCCAGAGC

*Note*: F, forward; R, reverse.

### 2.12 Western blot

Protein samples were harvested by lysing the cells using Radio‐Immunoprecipitation assay lysis solution (Beyotime). Subsequent to the measurement of protein concentration using the bicinchoninic acid kit (Beyotime), the corresponding volume of proteins was mixed with the loading buffer (Beyotime) and heated in a boiling‐water bath for 3 min for denaturation. The proteins underwent 30‐min electrophoresis at 80 V for 30 min and 1–2 h of electrophoresis at 120 V once the bromophenol blue entered the separation gel. Next, the proteins were transferred to membranes in an ice bath for 60 min with 300 mA current. After that, the membranes were rinsed for 1–2 min in the washing solution and sealed in the sealing solution for 60 min at room temperature or overnight at 4°C. The membranes were probed on a shaker at room temperature with primary antibodies against GLRX (PA5‐92389, 1:1000, Invitrogen), NRF1 (MA5‐32782, 1:1000, Invitrogen), METTL3 (PA5‐121190, 1:1000, Invitrogen), METTL14 (PA5‐117138, 1:1000, Invitrogen), and β‐actin (ab8226, 1:5000, Abcam) for 1 h, followed by three washes with the washing solution for 10 min each. Thereafter, the membranes were probed for 1 h with secondary antibodies (1:5000, Abcam) of goat anti‐rabbit immunoglobulin G (IgG, ab6702) or goat anti‐mouse IgG (ab6708) at room temperature before three washes for 10 min each. The membranes were added with developer solution, followed by detection with a chemiluminescent imaging system (Bio‐Rad).

### 2.13 Methylated RNA immunoprecipitation

A Magna MeRIP™ m6A kit (EMD Millipore) was employed for the methylated RNA immunoprecipitation (MeRIP) assay. Specifically, total RNA was extracted from pretreated cells or tissues and randomly split into 100 nucleotides. The RNA samples were then immunoprecipitated with magnetic beads pre‐coated with anti‐m^6^A antibody (EMD Millipore) or anti‐mouse IgG (EMD Millipore). The N6‐methyladenosine 5′‐monophosphate sodium salt was applied to elute m^6^A‐modified RNA fragments for further qRT‐PCR analysis. Specific primers for qRT‐PCR analysis were designed as per the SRAMP website (http://www.cuilab.cn/sramp/) analysis: F, 5′‐GCATCGCAGGATGTCAGTA‐3′; R, 5′‐CTGGATTTGGAAAACCTGGGC‐3′.

### 2.14 RNA immunoprecipitation

A Magna RIP RNA‐binding protein immunoprecipitation kit (17‐700, Sigma‐Aldrich) was employed for RNA immunoprecipitation (RIP) assays. After cells were lysed with RIP lysis solution, the whole cell lysates were incubated with IGF2BP2 antibodies (11601‐1‐AP, 1:50, Proteintech) and magnetic beads from the kit at 4°C, with IgG antibody (ab205718, 1:50, Abcam) as the NC. The beads were washed with pre‐cooled RIP Wash and incubated with proteinase K (10 mg/mL) to disrupt non‐specific binding. The immunoprecipitated RNA was purified with GLRX mRNA expression examined by qRT‐PCR.^25^

### 2.15 Chromatin immunoprecipitation

An EZ‐Magna ChIP TMA kit (17‐10,086, EMD Millipore) was adopted for the chromatin immunoprecipitation (ChIP) assay. Logarithmically growing BV‐2 cells were cross‐linked for 10 min with 1% formaldehyde, reacted with 125 mM glycine for 5 min at room temperature to terminate the cross‐linking, washed twice with pre‐chilled PBS, and centrifuged at 2000 *g* for 5 min, followed by cell collection. The collected cells were resuspended in cell lysis to a final concentration of 2 × 10^6^ cells per 200 μL. The resuspended cells were supplemented with protease inhibitor mixture, centrifuged at 5000 *g* for 5 min, resuspended in nuclear isolation buffer, lysed in an ice‐water bath for 10 min, and sonicated to attain 200–1000 bp chromatin fragments. The chromatin fragments were centrifuged at 14,000 *g* for 10 min at 4°C, followed by the obtaining of the supernatant. The supernatant (100 μL, DNA fragment) in each group was mixed completely with 900 μL ChIP dilution buffer, 20 μL protease inhibitor cocktail (50×), and 60 μL Protein A Agarose/Salmon Sperm DNA for 1 h at 4°C and then left to stand for 10 min at 4°C, followed by 1‐min centrifugation at 700 *g*. The supernatant (20 μL) was taken as the Input. The supernatant was supplemented with 1 μL NRF1 rabbit antibody (ab175932, Abcam) in the experimental group and 1 μL rabbit anti‐IgG (ab172730, Abcam) in the NC group. Each tube was supplemented with 60 μL Protein A Agarose/Salmon Sperm DNA, mixed well at 4°C for 2 h, and allowed to stand for 10 min. After 1‐min centrifugation at 700 *g* and the removal of the supernatant, the precipitate was washed with l mL low salt buffer, high salt buffer, LiCl solution, and tromethamine‐ethylene‐diamine tetra‐acetic acid buffer (twice), eluted twice with 250 μL ChIP Wash Buffer, and de‐cross‐linked with 20 μL NaCl (5 M) to recycle DNA. The enriched chromatin fragments were assayed by qRT‐PCR with METTL3 promoter primers: F, 5′‐AATCATGTGCATGCCTGGTG‐3′ and R, 5′‐CGCTCGTCCAGAAGACTCAG‐3′.

### 2.16 Dual‐luciferase assay

Dual‐luciferase reporter gene plasmids encompassing wild‐type (WT) and mutant (Mut, without high 3′‐untranslated region (UTR) confidence AAACT, Attachment S1) GLRX mRNA sequences, as well as METTL3 promoter WT (full‐length METTL3 promoter sequences) and Mut (METTL3 promoter sequence without the binding sites to NRF1, Attachment S2), were constructed, respectively. The reporter plasmids were co‐transfected into BV‐2 cells with si‐NC, si‐METTL3, or si‐NRF1, respectively. Subsequent to 24‐h transfection, the cells were lysed and centrifuged at 13,000 *g* for 1 min, followed by the harvesting of the supernatant. The luciferase activity was examined using a dual‐luciferase reporter gene assay kit (16,185, Thermo Fisher Scientific). Each cell sample was added with 100 μL Firefly luciferase working solution to detect Firefly luciferase activity and 100 μL Renilla luciferase working solution to measure Renilla luciferase activity (the internal reference). The ratio of Firefly to Renilla luciferase activity was adopted as the relative luciferase activity.

### 2.17 Statistical analysis

All experiments were repeated individually at least thrice. All data were stated as mean ± SD and analyzed using SPSS 13.0 software (SPSS Inc.). Comparisons between the two groups were analyzed using Student's *t*‐test, and comparisons among three or more groups were performed using one‐way analysis of variance and Tukey's post hoc test. *p* < 0.05 represented a statistically valuable difference.

## 3 RESULTS

### 3.1 GLRX expression was low in MPTP‐induced PD mice

**FIGURE 1:**
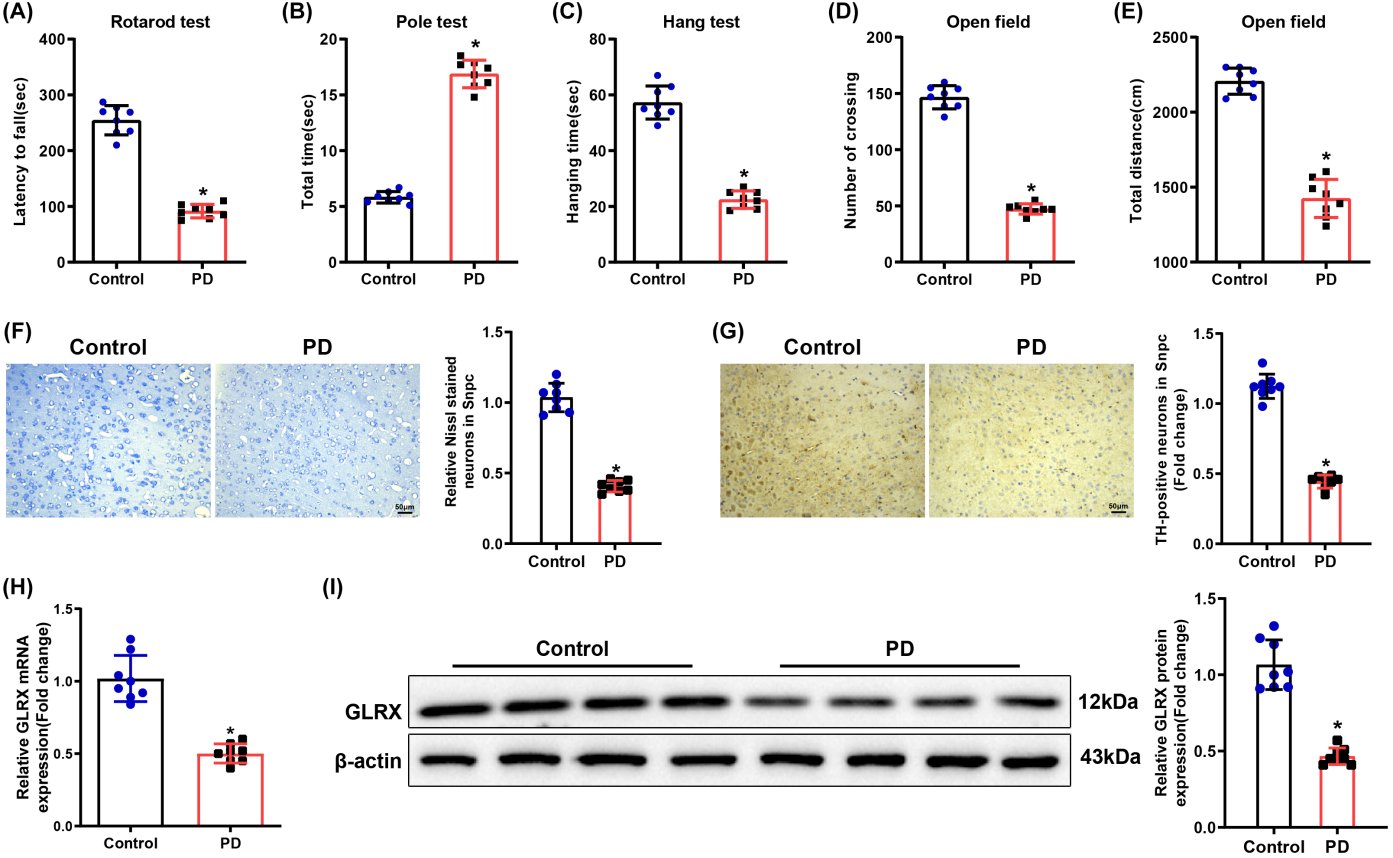
Low GLRX expression is observed in PD mice. (A) Residence time of mice on the rotarod in rotarod tests; (B) time required for mice to climb from the top of the pole down to the bottom (landing on both front paws) in pole tests; (C) hanging time of mice in hanging tests; (D) the number of crossings between the squares in open‐field tests; (E) total movement distance of mice in open‐field tests; (F) results of Nissl staining of mouse SNpc tissues; (G) immunohistochemistry detection of TH expression in mouse SNpc tissues; (H–I) qRT‐PCR and Western blot measurement of GLRX mRNA and protein expression in mouse SNpc tissues. The scale: 200×; data were displayed as mean ± SD, with *t*‐test for statistical analysis between the two groups. *p < 0.05 compared with the control group. *N* = 8. GLRX, glutaredoxin; PD, Parkinson's disease; SNpc, substantia nigra pars compacta; TH, tyrosine hydroxylase.

To dissect the impact of GLRX on PD, the PD mouse model was established by injecting mice with MPTP, with the motor coordination ability of mice tested by rotarod, pole, and open‐field tests, and the grip strength of forelimbs and hindlimbs examined by hanging test. Relative to control mice, MPTP‐injected mice exhibited a considerable reduction in time on the rotarod (Figure 1A), hanging time (Figure 1C), the number of crossings (Figure 1D), and total movement distance (Figure 1E) and an augmentation in the time of climbing the pole (Figure 1B). These results indicated that MPTP induced motor dysfunction in mice, which is a symptom of PD. The results of Nissl and immunohistochemistry staining demonstrated that the number of Nissl‐positive neurons and TH expression were remarkably reduced in mouse SNpc after MPTP injection (Figure 1F–G). Collectively, the MPTP‐induced PD mouse model was successfully established. The results of qRT‐PCR and Western blot disclosed that GLRX expression prominently decreased in the SNpc of MPTP‐induced PD mice (Figure 1H–I), suggesting that GLRX was poorly expressed in MPTP‐induced PD mice.

### 3.2 Overexpression of GLRX mitigated PD progression in mice

**FIGURE 2:**
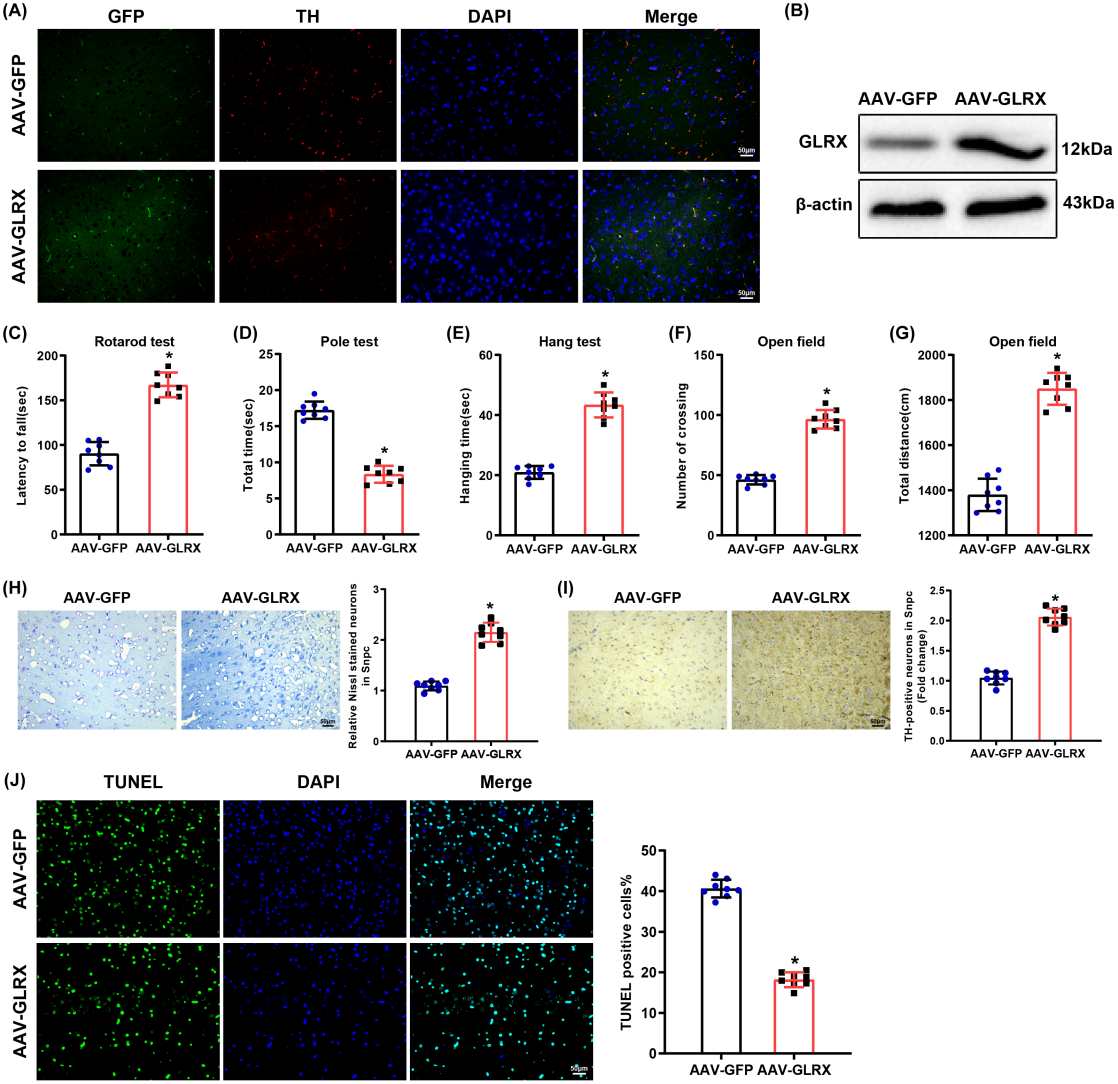
PD progression is attenuated via overexpression of GLRX in mice. The brains of PD mice were injected with viruses overexpressing GLRX using AAV‐GFP as the vector. (A) Immunofluorescence measurement of GFP and TH expression in SNpc tissues of PD mice, scale bar: 20 μm. (B) Western blot examination of GFP‐GLRX expression in SNpc tissues of PD mice; (C) residence time of mice on the rotarod in rotarod tests; (D) time required for mice to climb from the top of the pole down to the bottom (landing on both front paws) in pole tests; (E) hanging time of mice in hanging tests; (F) total movement distance of mice in open‐field tests; (G) the number of crossings between the squares of mice in open‐field tests; (H) results of Nissl staining of SNpc tissues of PD mice; (I) immunohistochemistry determination of TH expression in SNpc tissues of PD mice; (J) TUNEL assay for cell apoptosis in SNpc tissues of PD mice. The scale: 200×; data were displayed as mean ± SD, with *t*‐test for statistical analysis between the two groups. *p < 0.05 compared with the AAV‐GFP group. *N* = 8. AAV, adeno‐associated virus; GFP, green fluorescent protein; GLRX, glutaredoxin; PD, Parkinson's disease; SNpc, substantia nigra pars compacta; TH, tyrosine hydroxylase.

Viruses overexpressing GLRX with AAV‐GFP as the vector were injected into the brains of PD mice through stereotactic injection. The immunofluorescence assay displayed that there was GFP fluorescence in TH‐positive neurons in the SNpc of PD mice (Figure 2A). As depicted in Figure 2B, GFP‐GLRX protein was successfully expressed in the SNpc of MPTP‐induced PD mice. These results indicated a successful infection of mice with AAV‐GFP.

The data manifested that after overexpression of GLRX, the time spent on the rotarod (Figure 1C), hanging time (Figure 1E), the number of crossings (Figure 1F), and the total movement distance (Figure 1G) were appreciably augmented but the time spent on climbing the pole (Figure 1D) apparently declined in MPTP‐induced PD mice. After overexpression of GLRX, the number of Nissl‐positive neurons and TH expression were dramatically elevated in the SNpc of PD mice (Figure 2H–I). Moreover, the TUNEL assay demonstrated that overexpression of GLRX diminished cell apoptosis in the SNpc of MPTP‐induced PD mice (Figure 2J). Conclusively, overexpression of GLRX alleviated motor dysfunction and dopamine neuron degeneration in MPTP‐induced PD mice.

### 3.3 METTL3 promoted m^6^A modification of GLRX mRNA and enhanced IGF2BP2‐dependent stability of GLRX mRNA

**FIGURE 3:**
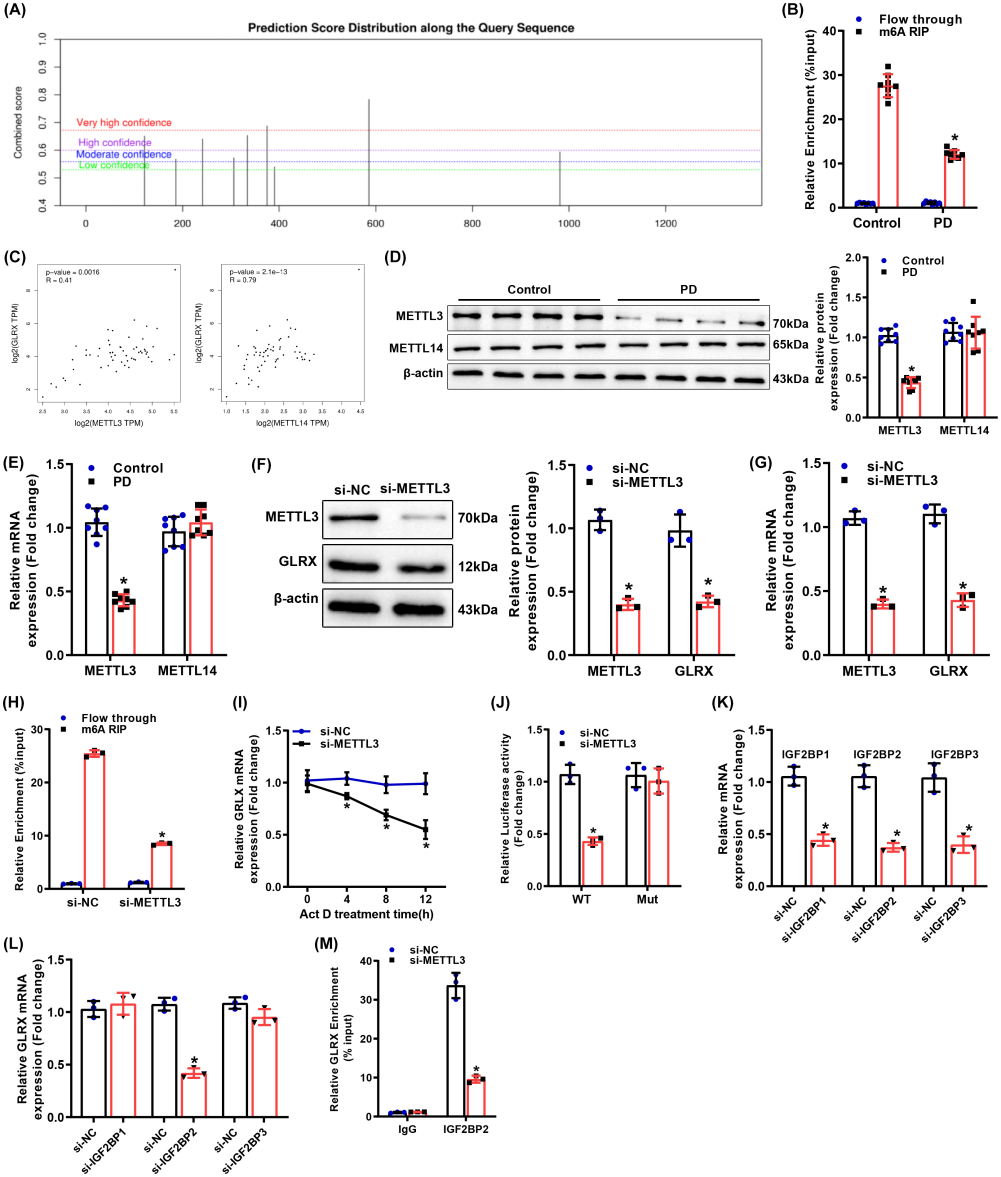
METTL3 promotes m^6^A modification and IGF2BP2‐dependent stability of GLRX mRNA. (A) SRAMP website prediction of the abundance of m^6^A methylation sites of GLRX; (B) MeRIP to evaluate the m^6^A methylation level of GLRX in SNpc tissues of PD mice; (C) GEPIA2 database to predict the correlation of GLRX expression with the expression of m^6^A methylation enzymes METTL3 and METTL14 in SNpc tissues; (D–E) qRT‐PCR and Western blot to assess the mRNA and protein expression of METTL3 and METTL14 in SNpc tissues of PD mice; (F–G) qRT‐PCR and Western blot to test METTL3 and GLRX mRNA and protein expression in BV‐2 cells after knockdown of METTL3. (H) MeRIP to measure the m^6^A methylation level of GLRX in BV‐2 cells. (I) qRT‐PCR to identify GLRX expression after treatment of BV‐2 cells with transcriptional inhibitor actinomycin D. (J) Dual‐luciferase assay to evaluate the binding relationship between METTL3 and GLRX; (K, L) qRT‐PCR determination of GLRX mRNA expression in BV‐2 cells; (M) RIP assay to determine the binding relationship between IGF2BP2 and GLRX in BV‐2 cells. *p < 0.05 compared with the Flowthrough, si‐NC, or control group. Data were displayed as mean ± SD, with *t*‐test for statistical analysis between the two groups and one‐way analysis of variance for statistical analysis among multiple groups, and Tukey's test for post hoc analysis. The cell experiments were all repeated thrice. For animal experiment, *N* = 8. GLRX, glutaredoxin; IGF2BP2, insulin‐like growth factor 2 mRNA‐binding protein 2; METTL3, methyltransferase‐like 3; NC, negative control; PD, Parkinson's disease; SNpc, substantia nigra pars compacta; TH, tyrosine hydroxylase.

The presence of methylation modification sites on GLRX mRNA sequences was identified using the m^6^A prediction website SRAMP (http://www.cuilab.cn/sramp), with the most likely modification site located in the 3′‐UTRs (Figure 3A, Attachment S1). (MeRIP)‐qPCR results depicted that m^6^A antibody markedly enriched GLRX mRNA in mouse SNpc versus IgG group but remarkably reduced GLRX mRNA enrichment in PD mice relative to control mice (Figure 3B). This result illustrated that m^6^A modification occurred in GLRX mRNA and that the m^6^A modification level of GLRX decreased in the MPTP‐induced PD mouse model.

Prediction by the GEPIA2 website (http://gepia2.cancer‐pku.cn/#index) revealed that in SNpc, GLRX expression was proportional to both m^6^A methylation enzymes METTL3 and METTL14 (Figure 3C). Western blot and qRT‐PCR identified low METTL3 expression and no apparent alteration in METTL14 expression in the PD model (Figure 3D–E). Therefore, we speculated that METTL3 might mediate the m^6^A modification of GLRX mRNA.

After knockdown of METTL3 in BV‐2 cells, GLRX expression was considerably diminished (Figure 3F–G), as was the m^6^A modification level of GLRX mRNA (Figure 3H). Considering that m^6^A modification positively regulates GLRX mRNA levels, it was tested whether m^6^A modification affected the stability of GLRX mRNA. qRT‐PCR assay was performed after treatment of cells with the transcriptional inhibitor actinomycin D, which exhibited that knockdown of METTL3 signally decreased the stability of GLRX mRNA (Figure 3I). Dual‐luciferase assay presented that knockdown of METTL3 evidently decreased the transcription levels of WT GLRX but did not affect the transcription levels of Mut GLRX (Figure 3J). In addition, we combined si‐METTL3 with the overexpression vector AAV‐GLRX to investigate the effect on GLRX expression in BV‐2 cells. The results showed that compared with the si‐METTL3 group, the si‐METTL3 + oe GLRX group showed a significant increase in GLRX expression, but no significant change in METTL3 expression (Figure S1). These results indicated that METTL3 promoted GLRX expression through m6A modification.

It was reported that IGF2BPs, including IGF2BP1/2/3, enhance mRNA stability and translation by recognizing m^6^A motifs to target thousands of mRNA transcripts.^26^ Therefore, three specific siRNAs were designed for IGF2BP1/2/3, and the results described that knockdown of IGF2BP2 conspicuously reduced GLRX mRNA expression, while knockdown of IGF2BP1 or IGF2BP3 had no prominent effect (Figure 3K,L). Moreover, IGF2BP2‐specific antibodies strikingly enriched GLRX mRNA, whereas knockdown of METTL3 noticeably diminished GLRX mRNA enriched by IGF2BP2‐specific antibodies (Figure 3M). Collectively, IGF2BP2 enhanced GLRX mRNA stability in an m^6^A‐dependent manner.

### 3.4 The transcription factor NRF1 bound to METTL3 promoter and promoted the transcriptional expression of METTL3

**FIGURE 4:**
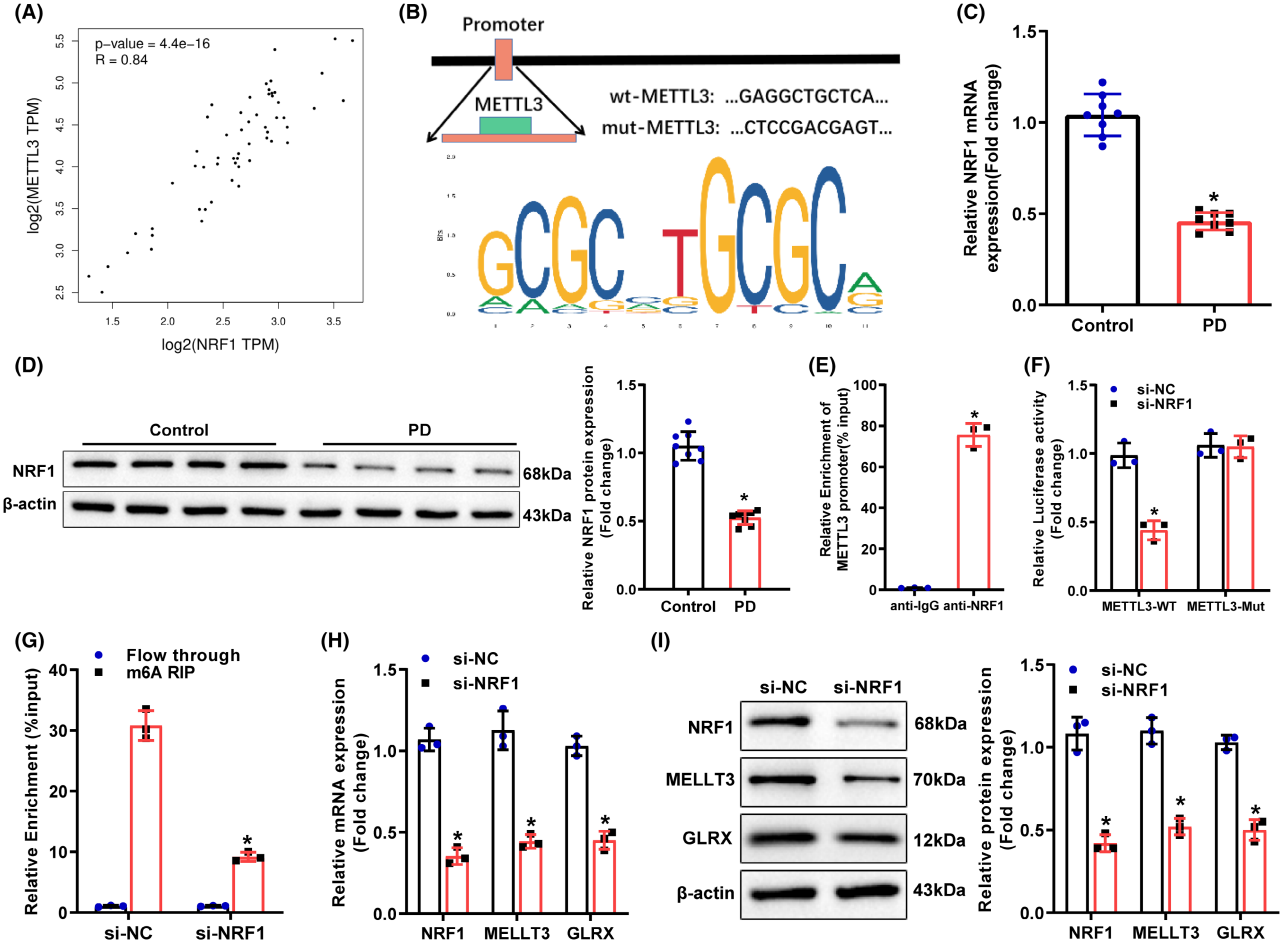
NRF1 binds to the METTL3 promoter to enhance METTL3 transcriptional expression. (A) GEPIA2 database prediction of the correlation between NRF1 expression and the m^6^A methylation enzyme METTL3 expression in SNpc tissues. (B) JASPAR CORE website analysis of the presence of binding sites of NRF1 to METTL3 promoter region. (C, D) qRT‐PCR and Western blot measurement of NRF1 mRNA and protein expression in SNpc tissues of PD mice. (E) ChIP assay to assess NRF1 enrichment in METTL3 promoter region; (F) dual‐luciferase assay to detect the targeting relationship between METTL3 and NRF1 in BV‐2 cells; (G) MeRIP to measure the m^6^A methylation level of GLRX in BV‐2 cells. (H–I) qRT‐PCR and Western blot to assess NRF1, METTL3, and GLRX mRNA and protein expression in BV‐2 cells, *p < 0.05 compared with the si‐NC, control, or anti‐IgG group. Data were displayed as mean ± SD, with *t*‐test for statistical analysis between the two groups and one‐way analysis of variance for statistical analysis among multiple groups, and Tukey's test for post hoc analysis. The cell experiments were all repeated thrice. For animal experiment, *N* = 8. GLRX, glutaredoxin; METTL3, methyltransferase‐like 3; NC, negative control; NRF1, nuclear factor erythroid 2‐like 1; PD, Parkinson's disease; SNpc, substantia nigra pars compacta.

Through the GEPIA2 website prediction, METTL3 expression was proportional to NRF1 expression in SNpc (Figure 4A). The analysis of the JASPAR CORE website (https://jaspar.genereg.net/) identified the target binding sites for NRF1 in the METTL3 promoter region (Attachment S2, Figure 4B). Therefore, we hypothesized that NRF1 might facilitate METTL3 transcriptional expression by binding to the METTL3 promoter.

As reflected by Western blot and qRT‐PCR, NRF1 expression was low in PD mice (Figure 4C,D). ChIP assay results documented that NRF1 was enriched in METTL3 promoter in BV‐2 cells (Figure 4E). Dual‐luciferase assay exhibited that knockdown of NRF1 evidently decreased the luciferase activity in METTL3 promoter WT but did not change the luciferase activity in METTL3 promoter Mut (Figure 4F). Meanwhile, knockdown of NRF1 strikingly decreased the expression of NRF1, METTL3, and GLRX and the m^6^A modification level of GLRX3 mRNA in BV‐2 cells (Figure 4G–I). Collectively, the transcription factor NRF1 was enriched in METTL3 promoter and elevated METTL3 transcriptional expression.

### 3.5 Knockdown of METTL3 reversed the alleviatory impact of NRF1 overexpression on motor dysfunction and dopamine neuron degeneration in PD mice

**FIGURE 5:**
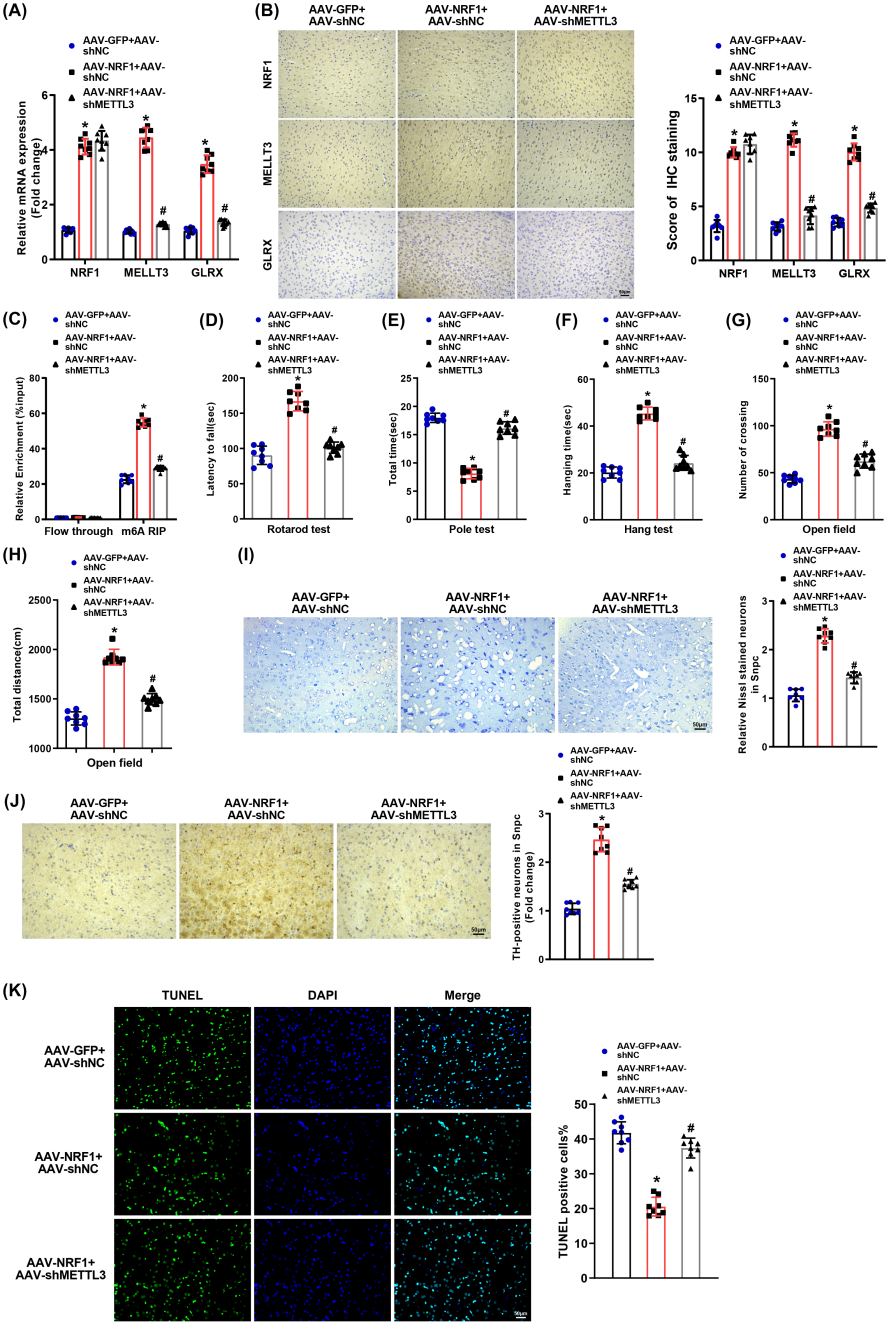
NRF1 elevates METTL3 expression to mitigate PD progression in mice. The brains of PD mice were injected with viruses overexpressing NRF1 or/and viruses knocking down METTL3 using AAV‐GFP as the vector. (A) qRT‐PCR detection of NRF1, METTL3, and GLRX mRNA expression in SNpc tissues of PD mice; (B) immunohistochemistry to test NRF1, METTL3, and GLRX expression in SNpc tissues of PD mice; (C) MeRIP examination of m^6^A methylation level of GLRX mRNA; (D) residence time of mice on the rotarod in rotarod tests; (E) time required for mice to climb from the top of the pole down to the bottom (landing on both front paws) in pole tests; (F) hanging time of mice in hanging tests; (G) the number of crossings between the squares of mice in open‐field tests; (H) total movement distance of mice in open‐field tests; (I) results of Nissl staining of SNpc tissues of PD mice; (J) immunohistochemistry to detect TH expression in SNpc tissues of PD mice; (K) TUNEL assay to measure cell apoptosis in SNpc tissues of PD mice. The scale: 200×. Data were displayed as mean ± SD, with one‐way analysis of variance for statistical analysis among multiple groups, and Tukey's test for post hoc analysis. *p < 0.05 compared with the AAV‐GFP + sh‐NC group and ^#^ *p* < 0.05 compared with the AAV‐NRF1 + sh‐NC group. For animal experiment, *N* = 8. AAV, adeno‐associated virus; GFP, green fluorescent protein; GLRX, glutaredoxin; METTL3, methyltransferase‐like 3; NC, negative control; NRF1, nuclear factor erythroid 2‐like 1; PD, Parkinson's disease; SNpc, substantia nigra pars compacta; TH, tyrosine hydroxylase.

To dissect the influence of NRF1‐mediated METTL3 expression on motor dysfunction and dopamine neuron degeneration in MPTP‐induced PD mice, viruses overexpressing NRF1 and knocking down MELLT3 were constructed to infect PD mice. qRT‐PCR and immunohistochemistry manifested that overexpression of NRF1 elevated NRF1, METTL3, and GLRX expression in PD mice, while further knockdown of METTL3 diminished METTL3 and GLRX expression in the presence of overexpression of NRF1 (Figure 5A,B). In addition, NRF1 upregulation augmented m^6^A modification of GLRX1 mRNA, which was abrogated by further knockdown of METTL3 (Figure 5C).

As depicted in Figure 5D–H, overexpression of NRF1 obviously augmented the time spent on the rotarod, hanging time, the number of crossings, and total movement distance while observably decreasing the time spent on climbing the pole. Additionally, NRF1 overexpression noticeably enhanced Nissl‐positive neuron number and TH expression but substantially reduced apoptotic cells in SNpc tissues of MPTP‐induced PD mice (Figure 5I–K). Nevertheless, further knockdown of METTL3 reversed the aforementioned phenomena (Figure 5D–K). Conclusively, overexpression of NRF1 ameliorated motor dysfunction and dopamine neuron degeneration in MPTP‐induced PD mice via promotion of METTL3 expression.

## 4 DISCUSSION

With no curable treatment and increasingly severe motor problems and non‐motor features that are resistant to treatment, PD is still a progressive disease that ultimately leads to serious disability.^27^ Therefore, the hunt for modification factors that result in disease progression and further alleviate the disease is an issue to be addressed in current and future research for the treatment of PD. The present study revealed that transcription factor NRF1 mitigated motor dysfunction and dopamine neuron degeneration in PD mice by elevating GLRX expression through the enhancement of METTL3 transcription.

A preceding study demonstrated that MPTP, a lipophilic molecule that can easily cross the blood–brain barrier, is the most frequently applied neurotoxin in animal models of PD.^28^ Nissl bodies and TH are markers of neurons and dopaminergic neurons, respectively.^29^ To explore the actions of modification factors on PD progression, a PD mouse model was first established by injecting MPTP into mice, with examination of the motor coordination ability of mice and the number of Nissl‐positive neurons and TH expression in mouse SNpc tissues.

GLRX is a redox protein in the thioredoxin family, which is increasingly essential in neurodegenerative disorders through mediation of neuroprotection from oxidative stress, promotion of mitochondrial function, and regulation of gene expression in the central nerve system.^30^ Gong et al. discovered low expression of GLRX in the midbrain tissue of PD patients and the SNpc of MPTP‐injected mice.^31^ Similar to their results, the present study also identified low expression of GLRX in the MPTP‐induced PD mouse model. Absence of GLRX has been reported to aggravate neurodegeneration in *Caenorhabditis elegans* PD models.^32^ Overexpression of GLRX1 substantially attenuates MPP^+^ (a toxic metabolite of MPTP that mediates neurodegenerative diseases)‐induced cell death.^33^ GLRX1 was reported to protect dopaminergic cells via an increase in protein glutathionization in the paraquat‐induced PD model.^34^ Verma et al. proposed that downregulation of GLRX1 in mice contributed to a diminution of TH‐ and Nissl‐positive neurons in mouse SNpc tissues, dopaminergic degeneration, and motor dysfunction in mice.^22^ Interestingly, the present study demonstrated consistent results that overexpression of GLRX augmented the number of Nissl‐positive neurons and TH expression of SNpc tissues and alleviated dopamine neuron degeneration and motor dysfunction in MPTP‐induced PD mice.

The m^6^A methylation process involves the transfer and removal of methylated groups through writers (methyltransferases) and erasers (demethylases) and the recognition of N6 methylation‐modified adenosine bases through readers (reading proteins), which activates downstream modulatory pathways.^35^ In our research, SRAMP predicted that methylation modification sites existed on GLRX mRNA sequences, and further (MeRIP)‐PCR elucidated that m^6^A modification occurred in GLRX mRNA. Changes in m^6^A modification levels and expression of related enzyme proteins may influence neuronal production, cerebral volume, memory and learning, and memory forming and consolidating, which is associated with the occurrence of disorders like PD, depression, Alzheimer's disease, epilepsy, and cerebral neoplasms.^14^, ^36^ Chen et al. uncovered that the decline in m^6^A leads to apoptosis of dopaminergic neurons.^15^ Concordantly, the present study elaborated that the MPTP‐induced PD mouse model had diminished the m^6^A modification level of GLRX. METTL3, the most well‐known m^6^A methyltransferase, is responsible for the reversible epi‐transcriptome manipulation of m^6^A modifications.^13^ Therefore, it could be speculated that METTL3 might orchestrate the m^6^A modification of GLRX mRNA. GEPIA2 website analysis displayed that GLRX expression was proportional to METTL3 in SNpc. Furthermore, dual‐luciferase assay and qRT‐PCR unraveled that GLRX mRNA stability was mediated by METTL3‐related m^6^A modifications. TIGF2BP family members are m^6^A readers.^37^ Our further experiments indicated that IGF2BP2 potentiated GLRX mRNA stability in an m^6^A‐dependent manner. Therefore, METTL3 promoted m^6^A modification of GLRX mRNA and increased IGF2BP2‐dependent stability of GLRX mRNA. Of note, a prior article illustrated that METTL3 mRNA was declined in the hippocampal tissue of postmortem patients with Alzheimer's disease,^16^ which corroborated our findings of METTL3 downregulation in MPTP‐induced PD mice. More importantly, ectopic METTL3 obviously facilitates long‐term memory formation.^38^ METTL3 silencing results in neuronal death in Alzheimer's disease in vivo and in vitro.^39^ Similarly, our results also documented that METTL3 knockdown decreased the number of Nissl‐positive neurons and TH expression in SNpc tissues and accelerated dopamine neuron degeneration and motor dysfunction in MPTP‐induced PD mice.

NRF1 is essential for maintaining protein and lipid homeostasis and cellular redox and takes charge of coordinated gene expression of all proteasome subunits when proteasomes are impaired in mammalian cells.^40^ Proteasome activity is necessary to maintain normal neurological function and defective activity of proteasome results in neurodegenerative disorders.^41^ Lee et al. noted that deletion of NRF1 in the brain caused altered proteasome genes and neurodegeneration.^18^ A study identified a low NRF1 expression in SNpc tissues of PD mouse models.^42^ Similarly, the present study elaborated that NRF1 expression was poor in MPTP‐induced PD mice. NRF1 participates in the promotion of mitochondrial biogenesis in human dopaminergic neuronal cells.^43^ It was documented that specific deficiency of NRF1 in the central nervous system leads to dysfunction of progressive motor neuron.^44^ Concurrent with these findings, our data elucidated that overexpression of NRF1 alleviated motor dysfunction and dopamine neuron degeneration in MPTP‐induced PD mice. However, there is no research on the relationship between NRF1 and METTL3. JASPAR CORE predicted the binding sites between METTL3 and NRF1, and subsequent experiments revealed that NRF1 boosted METTL3 transcription by binding to the METTL3 promoter. More importantly, the ameliorating impacts of NRF1 upregulation on motor dysfunction and dopamine neuron degeneration in MPTP‐induced PD mice were nullified by further knockdown of METTL3.

## 5 CONCLUSION

In conclusion, NRF1 relieved motor dysfunction and dopamine neuron degeneration in MPTP‐induced PD mice by increasing m^6^A modification of GLRX mRNA through elevation of METTL3 transcription. This study is hopeful to propose novel insights and theoretical basis for the future treatment of PD.

## FUNDING INFORMATION

Thanks for the grant from the Natural Science Foundation of Hunan Province (no. 2023JJ60298).

## CONFLICT OF INTEREST STATEMENT

The authors declare there is no conflict of interests.

## Supporting information


Figure S1.

Figure S2.

Table S1.

Table S2.

## Data Availability

The datasets used or analyzed during the current study are available from the corresponding author on reasonable request.
