# Supplementary figures and images for: NRF1 mitigates motor dysfunction and dopamine neuron degeneration in mice with Parkinson's disease by promoting GLRX m6A methylation through upregulation of METTL3 transcription
Source: CNS Neurosci Ther. 2023 Sep 22;30(3):e14441. doi: 10.1111/cns.14441 (PMC10916419; doi:10.1111/cns.14441)

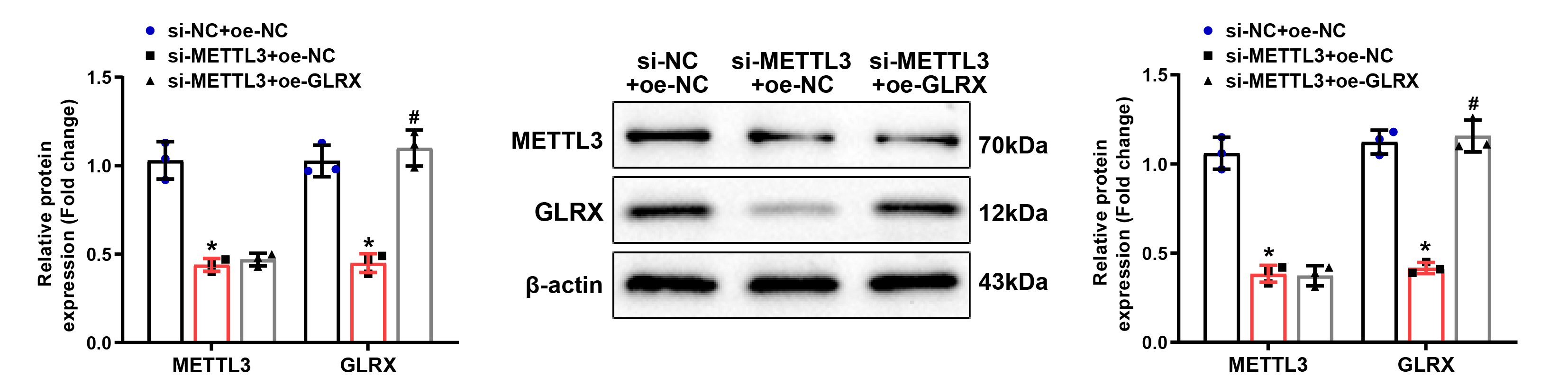

Supplement: Supplementary file 1 — Figure S1. [file CNS-30-e14441-s002.tif]
