# Supplementary material for: NRF1 mitigates motor dysfunction and dopamine neuron degeneration in mice with Parkinson's disease by promoting GLRX m6A methylation through upregulation of METTL3 transcription
Source: CNS Neurosci Ther. 2023 Sep 22;30(3):e14441. doi: 10.1111/cns.14441 (PMC10916419; doi:10.1111/cns.14441)

The original image of westernblot

Full unedited gel/blot for Figure 1I β-actin

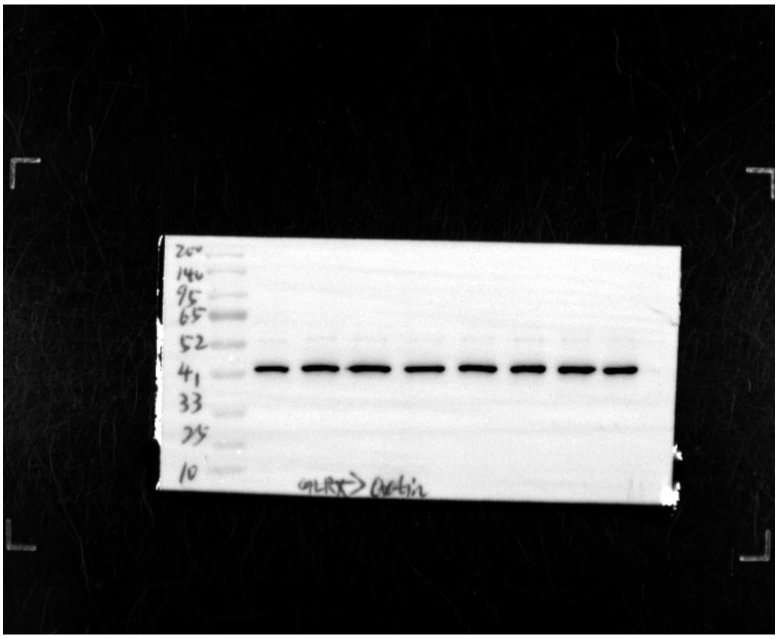

Full unedited gel/blot for Figure 1I GLRX

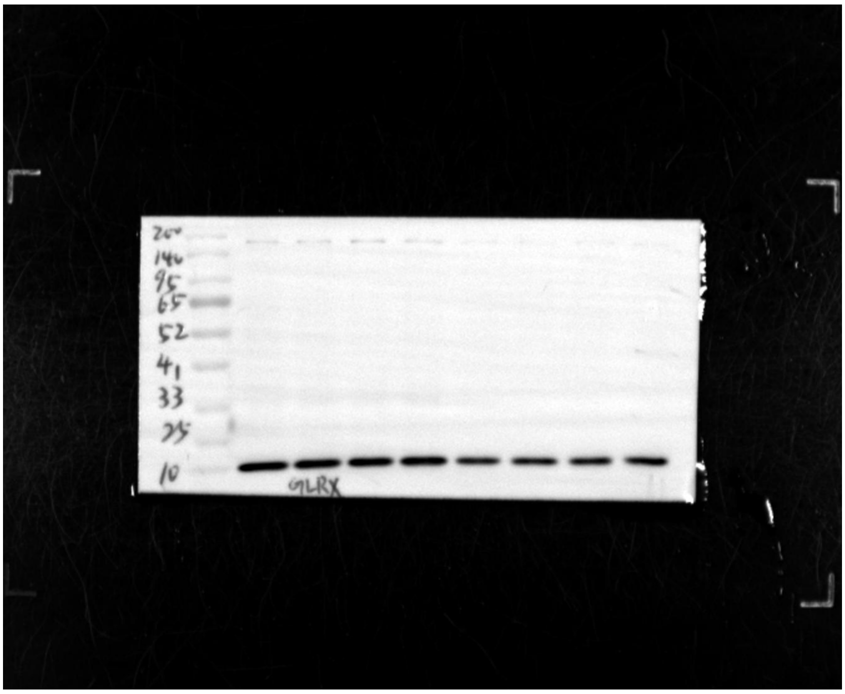

Full unedited gel/blot for Figure 2B β-actin

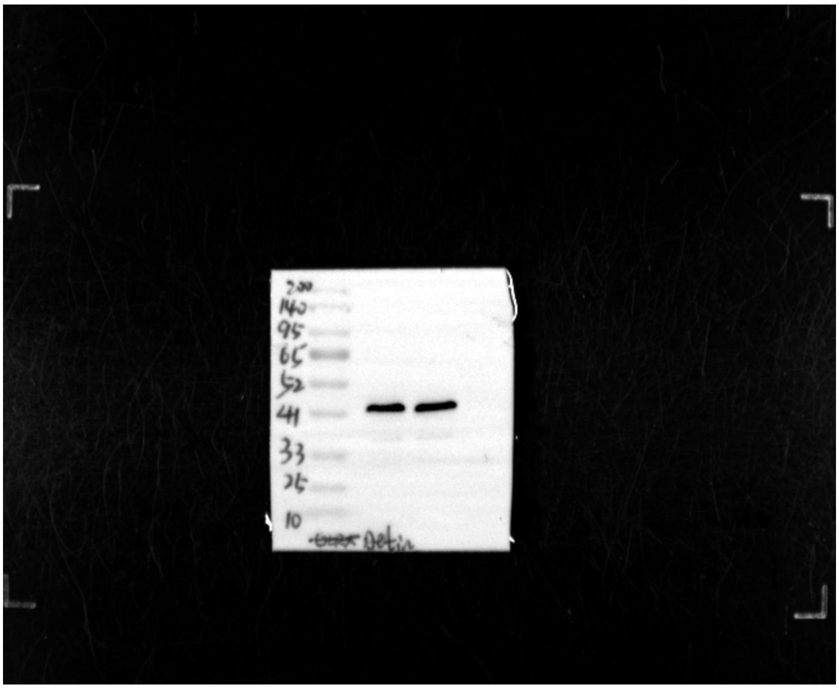

Full unedited gel/blot for Figure 2B GLRX

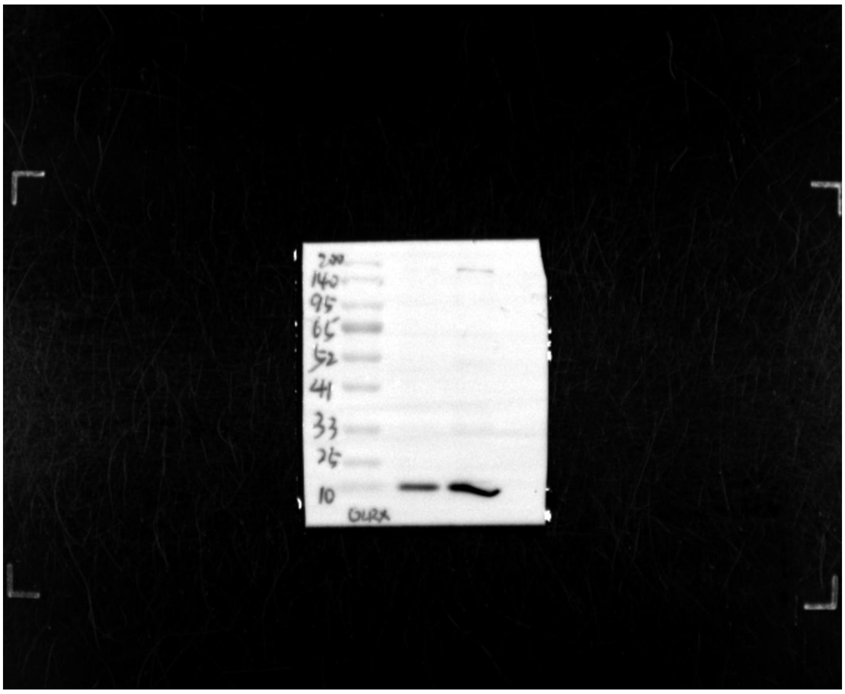

Full unedited gel/blot for Figure 3D β-actin

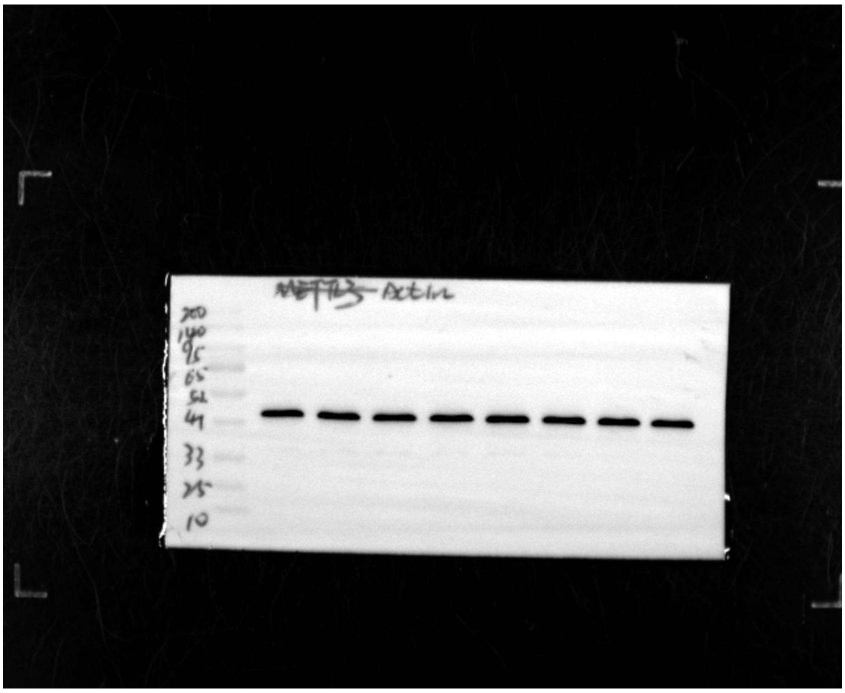

Full unedited gel/blot for Figure 3D METTL3

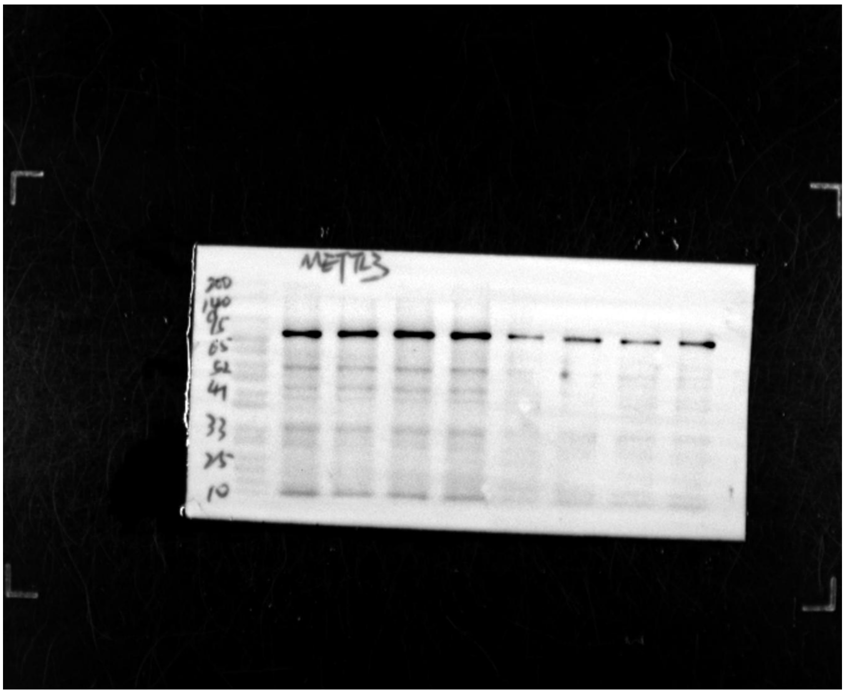

Full unedited gel/blot for Figure 3D METTL14

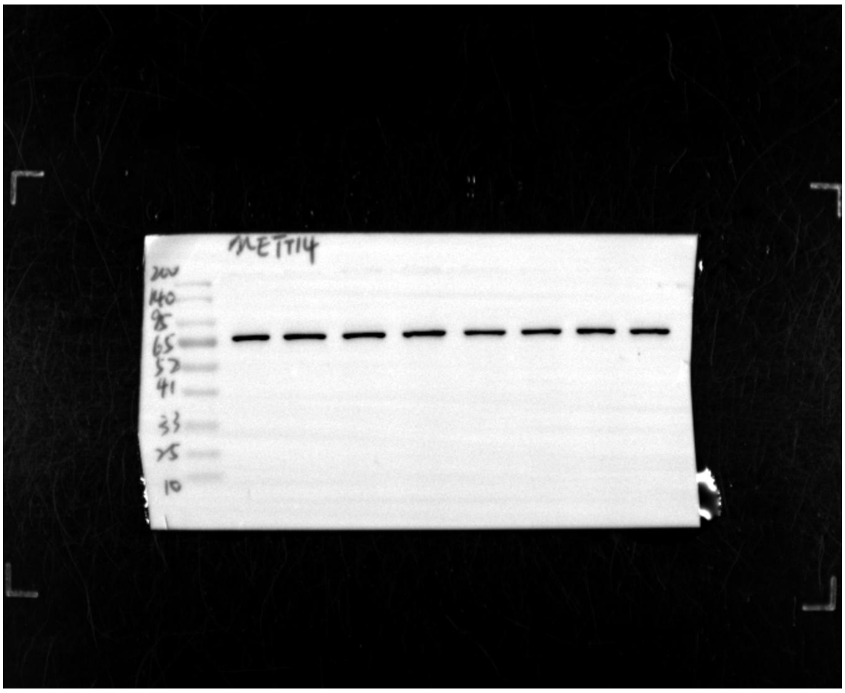

Full unedited gel/blot for Figure 3F β-actin

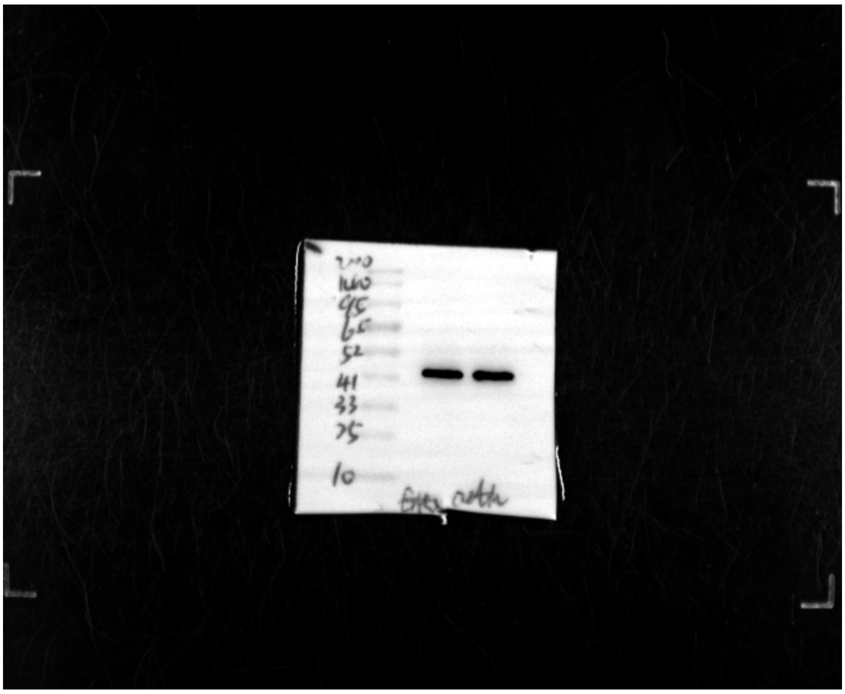

Full unedited gel/blot for Figure 3F GLRX

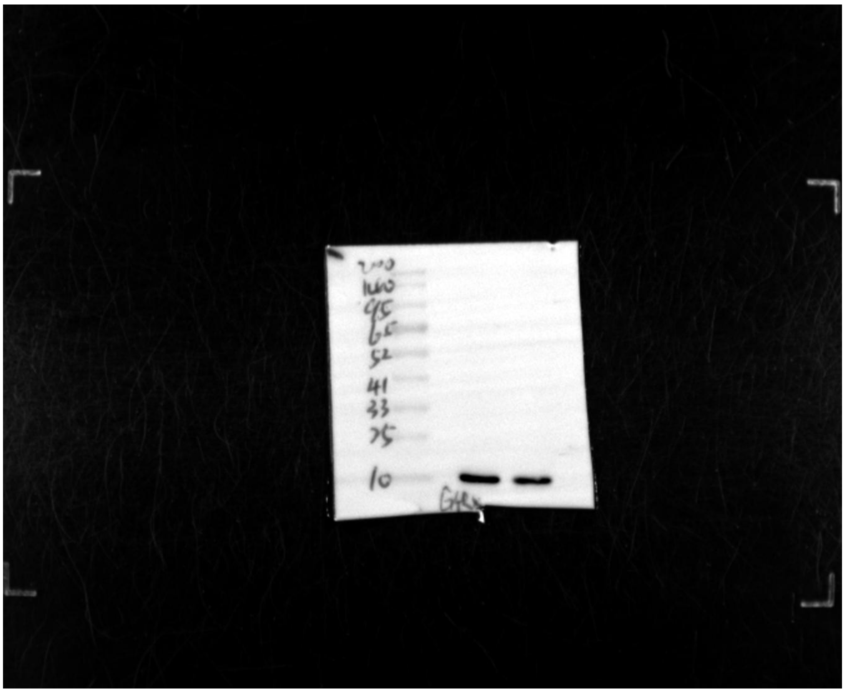

Full unedited gel/blot for Figure 3F METTL3

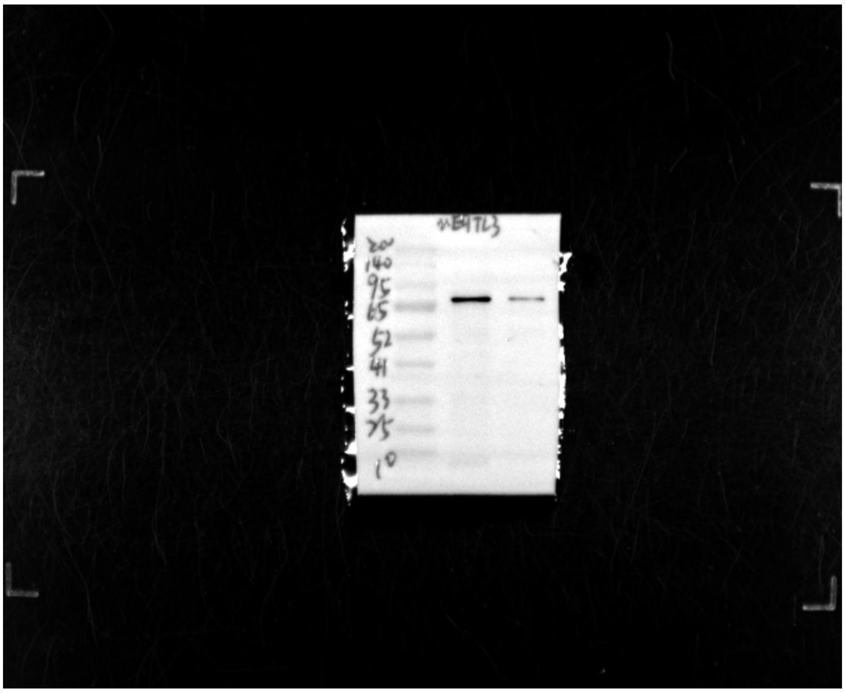

Full unedited gel/blot for Figure 4D β-actin

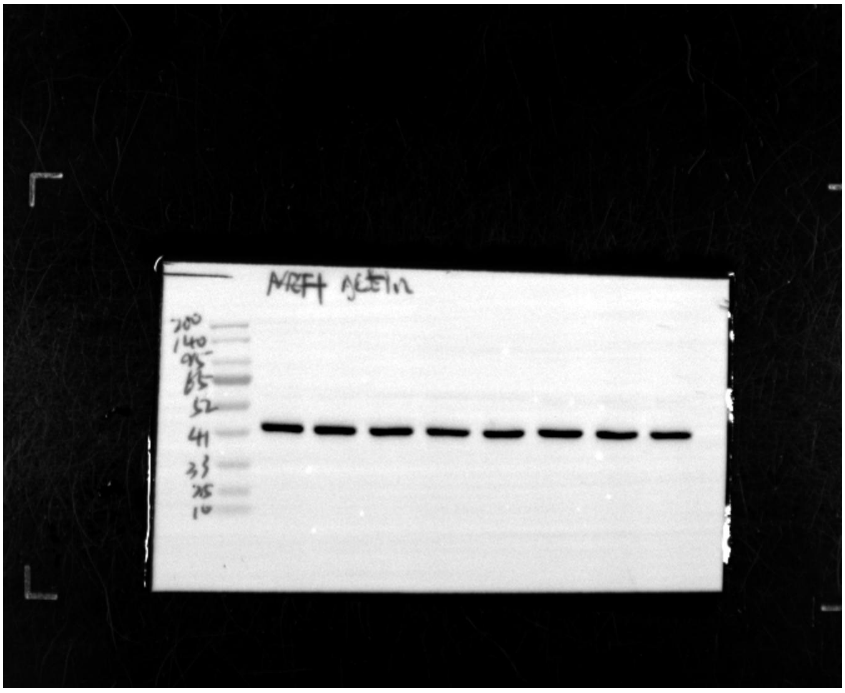

Full unedited gel/blot for Figure 4D NRF1

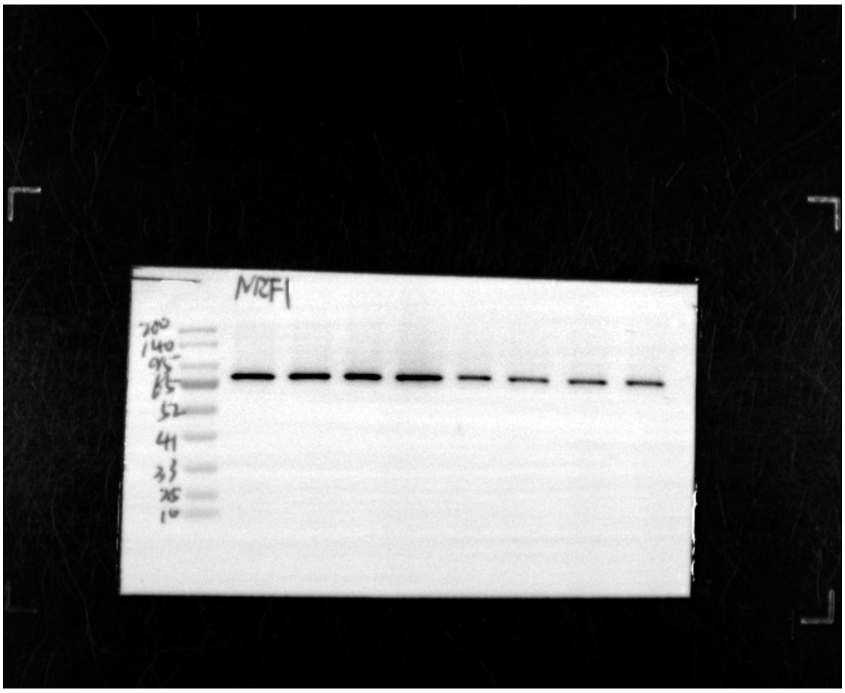

Full unedited gel/blot for Figure 4I β-actin

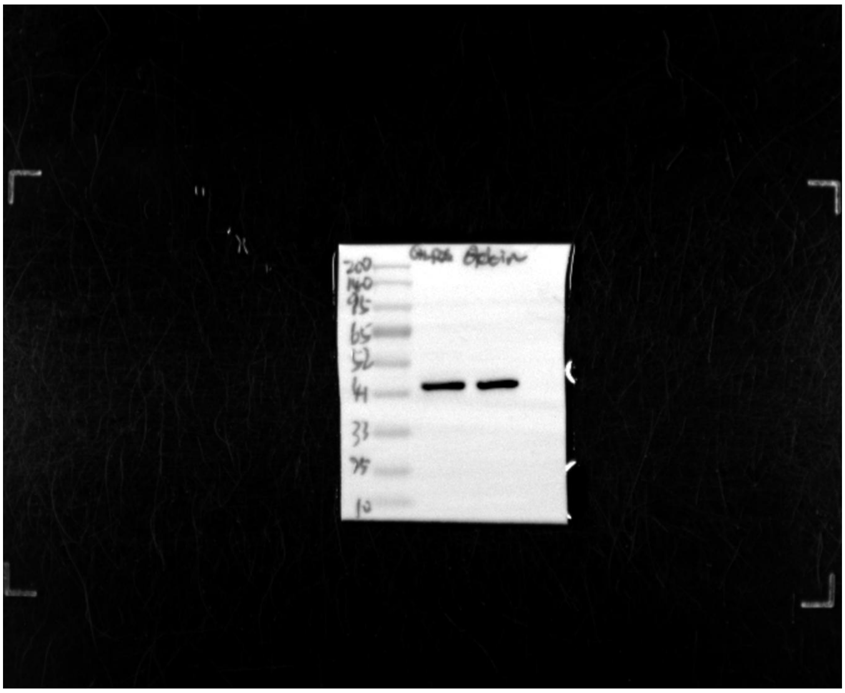

Full unedited gel/blot for Figure 4I GLRX

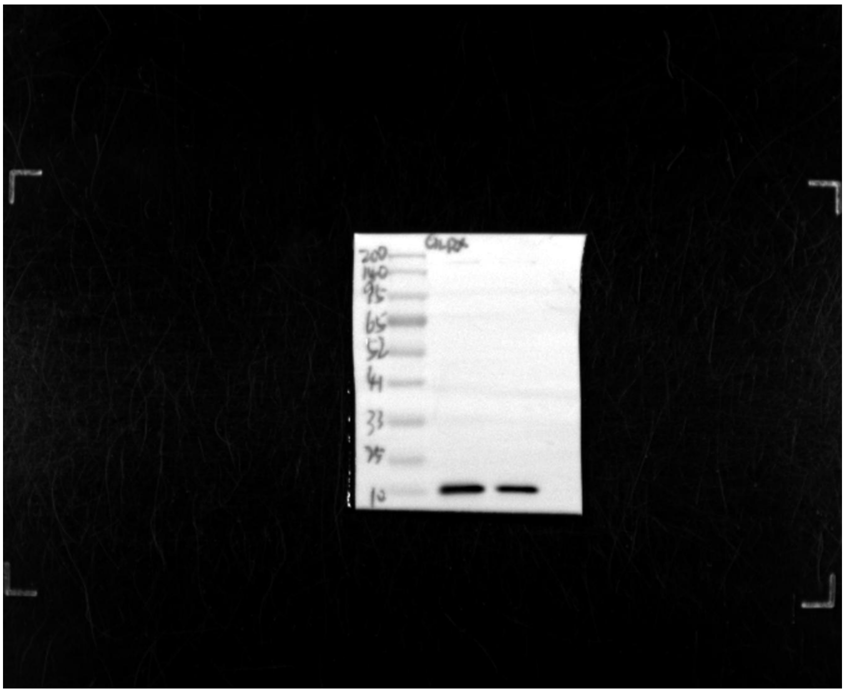

Full unedited gel/blot for Figure 4I METTL3

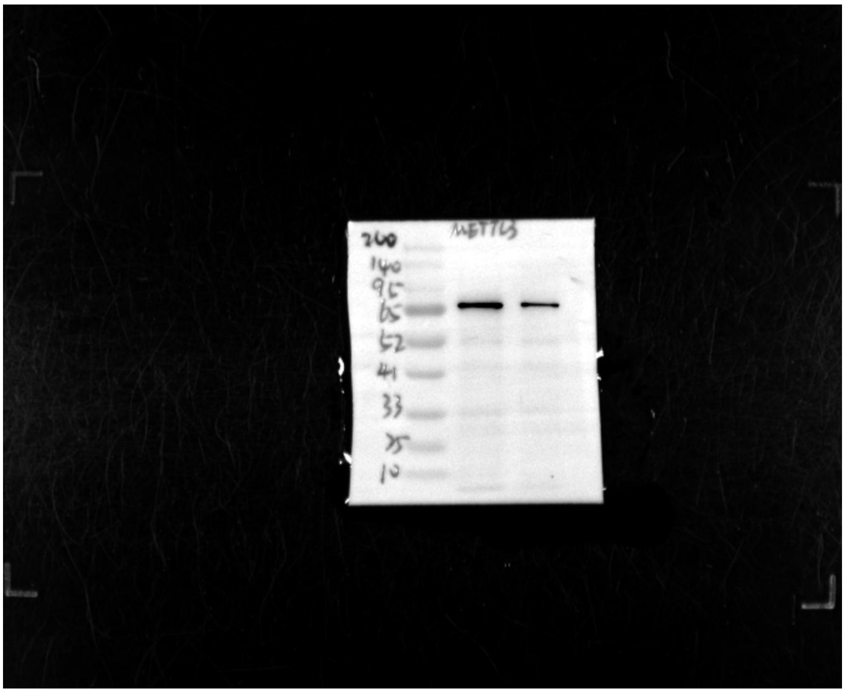

Full unedited gel/blot for Figure 4I NRF1

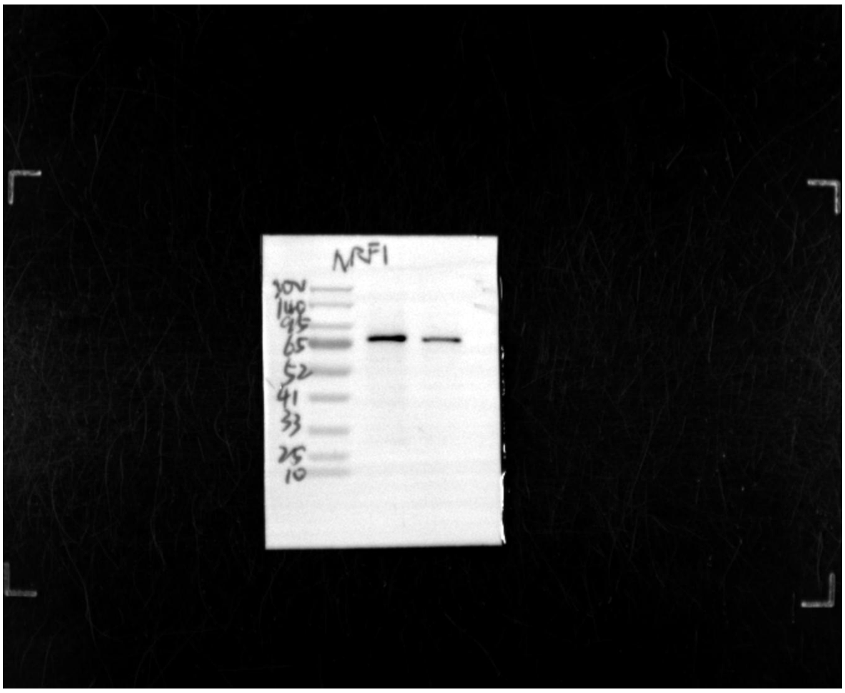

Full unedited gel/blot for Attached Figure 1 β-actin

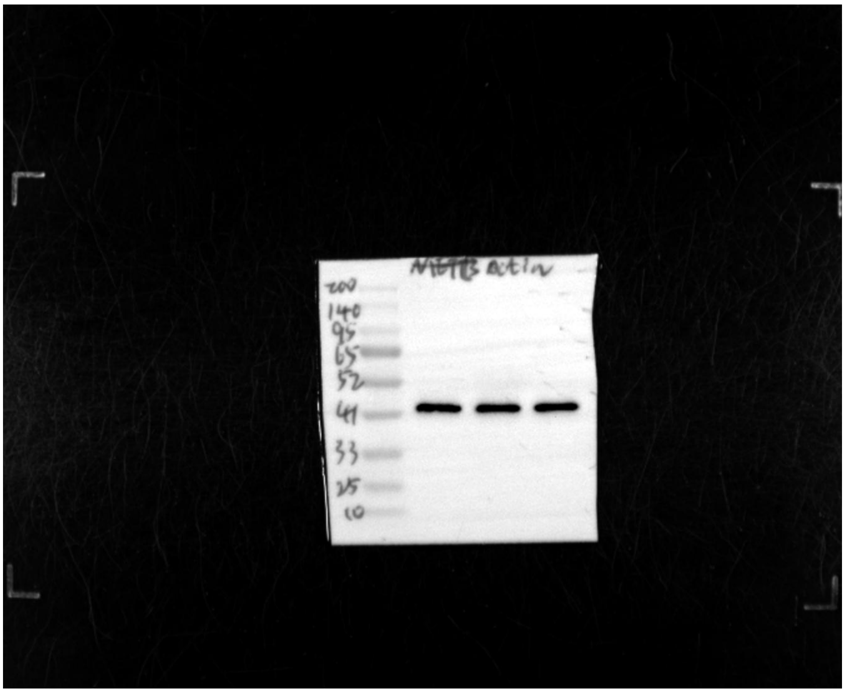

Full unedited gel/blot for Attached Figure 1 METTL3

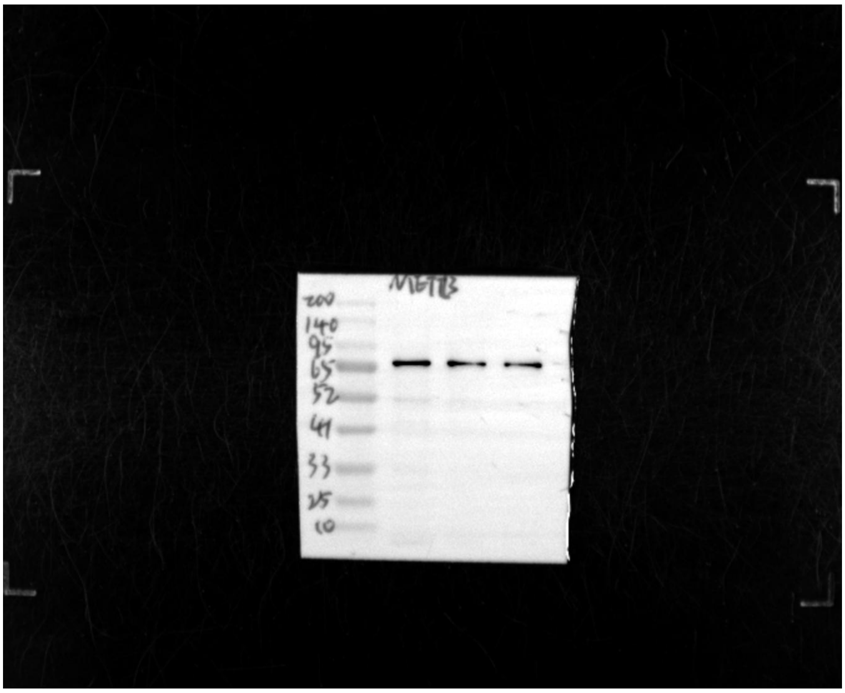

Full unedited gel/blot for Attached Figure 1 GLRX

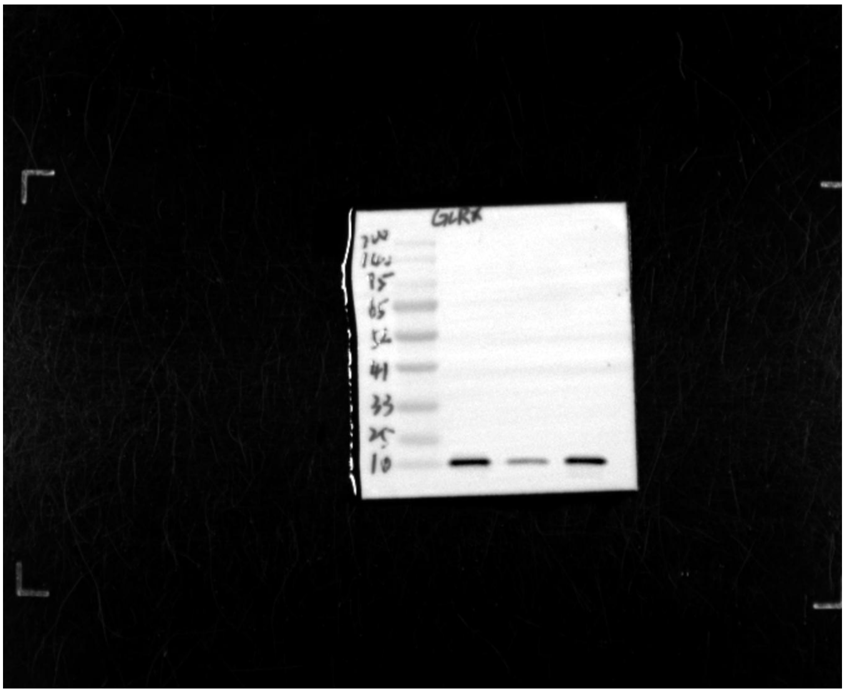

Supplement: Supplementary file 2 — Figure S2. [file CNS-30-e14441-s001.pdf]
